# Current State and Progress of Research on the Role of lncRNA in HBV-Related Liver Cancer

**DOI:** 10.3389/fcimb.2021.714895

**Published:** 2021-11-18

**Authors:** Xueke Wang, Meisong Kang, Chun Liu, Ting Lin, Xiao Han, Xiwen Jiang

**Affiliations:** ^1^ College of Biological Science and Engineering, Fuzhou University, Fuzhou, China; ^2^ DAAN Gene Co., Ltd. of Sun Yat-sen University, Guangzhou, China

**Keywords:** lncRNA, hepatocellular carcinoma, hepatitis B virus, HBx protein, microRNA

## Abstract

Hepatocellular carcinoma (HCC) is a malignant tumor with the highest mortality rate in the world, and hepatitis B virus (HBV) plays an important role in its development. Long noncoding RNA (lncRNA) is highly related to the inactivation of tumor suppressor genes and the activation of oncogenes in HCC. Researchers have used high-throughput sequencing technology to identify many noncoding transcripts related to the development of HCC and have studied the interaction between these transcripts and DNA, RNA, or protein to determine the relevant mechanism in the development of HCC. In general, the research on lncRNA represents a new field of cancer research, and the imbalance in lncRNA plays an pivotal role in the occurrence of liver cancer. In this review, we summarize some of the dysfunctional lncRNAs in human HCC associated with HBV infection. Their regulatory pathways, functions, and potential molecular mechanisms in the occurrence and development of HCC are discussed.

## 1 Introduction

Hepatitis B caused by the hepatitis B virus (HBV) is a major global public health problem (Parkin et al., 2005; Ghouri et al., 2017). According to the latest report from the World Health Organization, about 300 million people are infected with HBV (Parkin, 2006; Wang and Li, 2013; Bray et al., 2018). At the same time, hepatocellular carcinoma (HCC) represents the fourth leading and fastest rising cause of cancer death (841,000 new cases annually) and about 1 million people die from liver cirrhosis and HCC every year (Kennedy et al., 2018; Chen et al., 2019). More importantly, the occurrence of HCC is largely related to HBV infection and chronic hepatitis (Maule and Merletti, 2012; Fidler et al., 2017; Wang et al., 2019; Zhou et al., 2019; El-Khazragy et al., 2020). Therefore, it is very important to study the potential pathogenesis of HBV in hepatocarcinogenesis to provide accurate information for early screening, clinical diagnosis, targeted molecular therapy, and patient prognosis (Hoppe-Seyler and Hoppe-Seyler, 2011; Lichinchi et al., 2016; Li et al., 2016; Machida, 2020). In recent years, researches on long noncoding RNA (lncRNA) has been gradually increasing (Li et al., 2016; Qiu et al., 2017; Wong et al., 2018). While, LncRNA was usually defined as RNA with little capacity to code protein (Wang and Li, 2013). LncRNA is a non-protein-coding transcript widely involved in biological and physiological processes. It regulates gene expression at the epigenetic, transcriptional, and posttranscriptional levels. Using high-throughput sequencing analysis, researchers have identified a large number of noncoding transcripts and some regulation mechanisms of them. They have found that a variety of lncRNAs are involved in regulating the proliferation, migration, and invasion of HCC cells (Hu et al., 2019; TMK et al., 2019; Martínez-Barriocanal et al., 2020). There is evidence shows that lncRNA may be a competing and specific endogenous small RNA (ceRNA), which regulating the corresponding downstream targets, accompanying affecting the development of liver cancer cells (Jiang and Liu, 2018; Derderian et al., 2019; Zhou et al., 2019). Recently, with more studies have been launched,more evidences showing the crucial role of HBx in HBV-rlated HCC. The HBx also be called non-structural X protein, which encoded by one of the four overlapping open reading frames of the HBV gene. The statistics show that the present of HBx can regulate the expression of HCC-related lncRNAs, which has directly linked the HBV infection with HCC. In addition, lncRNA in the plasma of patients with liver cancer also has great potential to predict the occurrence of early liver cancer (Saus et al., 2016). Therefore, in this paper we describing a series of representative lncRNAs that have been thoroughly studied with regard to their mechanisms of action. Their mechanisms, mode of regulation, and regulatory paths were summarized in detail. This review provides a scientific basis for further study of the HCC-related pathogenesis of HBV-related liver cancer and the prognosis of patients with liver cancer and expands possibilities for the development of treatments for HBV-related liver cancer.

**Figure 1:**
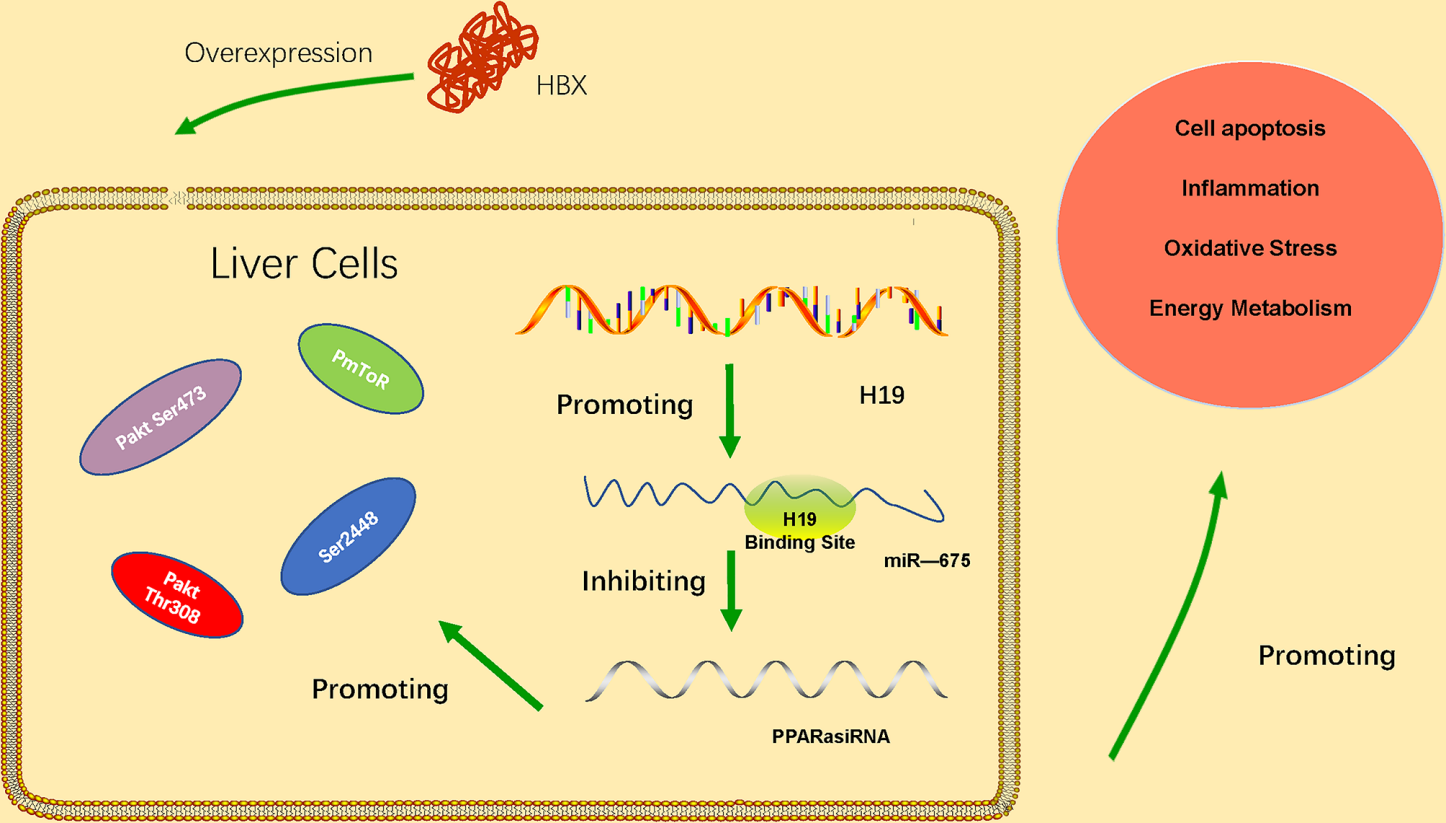
LncRNAs listed in this paper has been divided into three parts by their biological functions. The first part mainly introduce the lncRNAs which can advance or inhibit the proliferation of HCC cells; and second part shown the lncRNAs play a crucial role in HCC migration and invasion; and the last part present the lncRNAs abnormal expressing in the serum of HCC patients.

We have listed a series HCC-related lncRNAs reported by the latest studies, and dividing those lncRNAs into three parts by their biological functions. The first part mainly introduce the lncRNAs which can advance or inhibit the proliferation of HCC cells, and second part shown the lncRNAs play a crucial role in HCC migration and invasion;and the last part present the lncRNAs abnormal expressing in HCC patients’ serum. at the end of each parts we have given our own perspectives for those studies. And the logic of our classification has been shown in detail in **Figure 1**.

## 2 LncRNAs Related to HCC Proliferation

LncRNA can regulate the biological behavior of liver cancer cells in a variety of ways, such as activating immune lymphocytes to trigger inflammatory response and targeting key miRNAs to affect the expression level of downstream genes. A lot of studies have shown that many of those lncRNAs can be regulated by the HBV-encoded X protein (HBX), it is that the interactions between HBX HCC-related lncRNAs cause various of biological behavior of the HCC cells like proliferation, invade, apoptosis and autophagy. Among them, various pathways related to the development of liver cancer are involved, especially the major pathways and cytokines related to the proliferation of liver cancer cells, among which the major pathways involved are p53, Cal, PI3K-Akt, TGF-β and MAPK pathways. The expression levels of a series of key genes in these pathways, such as TLR9, STAT3, p53, TGF-β, and EZH2, have been reported to be regulated by lncRNAs, thus further affecting the proliferation of HCC cells and promoting the development of HCC.

### 2.1 LncRNAs Promote HCC Proliferation

#### 2.1.1 LncRNA FTX

FTX, a non-protein-coding gene on the human X chromosome, which has been characterized as a conserved transcript located at the inactivation center of the X chromosome. In addition to encoding two clusters of RNA tiny molecules, FTX encodes a highly conserved transcript consisting of 2356 nucleotides, lncRNA FTX, a recent study found that the expression of miR-545/374a clusters located in lncRNA FTX is positively regulated by HBV infection and may be induced by HBx expression (Zhao et al., 2014). The expression of miR-545/374a clusters was significantly upregulated in HBV-related HCC tissue, promoting the proliferation, migration, and invasion of HCC cells and being associated with a poor prognosis in HCC patients. The molecular mechanism of miR-545 and lncRNA FTX in HBV-associated cirrhosis has been explored (Liu et al., 2018). An important regulatory pathway has been discovered and confirmed in which lncRNA FTX/microRNA-545 regulates Tim-3 expression and affects the secretion of multiple inflammatory factors mainly by binding to ligand galactagglutinin-9 (galectin-9). This may inhibit CD8+ T-cell function in patients with chronic hepatitis B (CHB). Tim-3 is a potential target of miR-545, and miR-545 binding sites are located in the 3′-untranslated region (3′-UTR) of Tim-3 messenger RNA (mRNA). Liu et al. confirmed that lncRNA FTX mediates the regulation of Tim-3 gene transcription by negatively regulating microRNA-545 expression and affecting the secretion of monocytes to inflammatory cytokines, thus confirming that lncRNA FTX/microRNA-545/Tim-3 is involved in the inflammatory response process of hepatitis B cirrhosis. Furthermore, the expression of lncRNA FTX is upregulated in HCC tissue and is closely related to the degree of differentiation, metastasis, and envelope integrity of tumor tissue. The pathway of lncRNA FTX/microRNA-545 demonstrating a new perspective to study the relationship between lncRNA and HCC which has involved in galectin-9, a crucial protein in immune system, and also expound the mechanism that lncRNA FTX regulates the dysfunction of T-cell. Previous studies have presented a new perspective to research the relationship between lncRNAs and inflammatory.

#### 2.1.2 SFMBT2

In YılmazSusluer’s study (Yılmaz Susluer et al., 2018), they analyzed 135 lncRNAs in plasma samples of inactive carriers and 82 patients with resolved chronic HBV within 12 months of diagnosis and treatment. They mainly investigated the effects of small interfering RNA (siRNA)-mediated lincRNA-SFMBT2 silencing. Their study has transfected chemically synthesized siRNA into HCC cell lines, and the HBV DNA in transfected cells were detected by real-time PCR and ELISA, respectively HBsAg and HBeAg. Changes in lncRNA expression were observed in all three HBV groups compared to a control group. In patients with chronic HBV,the resistance to the expression of lincRNA-SFMBT2 and Zfhx2as increased significantly, while, lncRNA-Y5 expression has decreased. Moreover, decreased Y5 expression and increased lincRNA-SFMBT2 expression were observed in inactive HBsAg carriers. siRNA mediated inhibition of lincRNA-SFMBT2 has reduce the level of HBV DNA in human hepatoma cells. Further studies are needed to confirm the prognostic and therapeutic roles of these lncRNAs in HBV patients.

#### 2.1.3 HUR1

LncRNA HUR1 has been found significantly upregulated in HepG2-4D14 cells by using RNA deep sequencing to quantify the abundance of lncRNA in cells and HBV transgenic cells (Liu et al., 2018). In addition, experiments have shown that HBV-encoded X protein (HBX) enhances lnc-HUR1 transcription. Meanwhile, the overexpression of lnc-HUR1 can promotes cell proliferation has also been demonstrated. In contrast, knocking down the expression of lnc-HUR1 can inhibit cell growth. lnc-HUR1 interacts with p53 to inhibit transcriptional regulation of downstream genes such as p21 and B-cell lymphoma 2 related X proteins. Experiments showed that lnc-HUR1 levels in cells expressing HBx were significantly higher than those in cells transfected with pCMV FLAG-HBc or control plasmids, which means the presentation of HBx may promote the expression of HUR1. The lnc-HUR1 sequence was predicted by RNA Fold software. Since the researchers generated lnc-HUR1 promoter reporter genes, and as shown in the luciferase assay, HBx can significantly enhance lnc-HUR1 promoter activity. These results suggest that lnc-HUR1 is an HBV-related lncRNA and can be upregulated by HBx. HBV-upregulated lncRNA-HUR1 promotes cell proliferation and tumorigenesis by blocking p53 activity. LncRNA HUR1 may play an important role in HBV-related HCC and can be used as a therapeutic marker for HCC.

#### 2.1.4 LINC01152

LINC01152, a newly discovered long intergenic lncRNA located on chromosome 17 q24.3, plays a very important role in the occurrence and development of HBV-associated HCC. Expression of LINC01152 in HBV-positive liver cancer tissue and cells is significantly increased and can be induced by HBx *in vitro* (Chen et al., 2019). The HBx could increase the expression of LINC01152 by upgrading the transcription of LINC01152. Elevated LINC01152 can bind to the promoter region of IL-23, promoting its transcriptional activity and upregulating the protein levels of Stat3 and p-Stat3. IL-23, a newly discovered proinflammatory cytokine, helps maintain and expand Th17 cells and has been proved that could promotes tumorigenesis and development in HCC. These findings highlight the important role of HBx-LINC01152/IL-23 axis signaling in the proliferation of liver cancer and clarify the mechanism through which HBx protein could promote the proliferation of hepatoma cells by inducing the HBx-LINC01152 axis which demonstrates the important role of HBx in LINC01152/IL-23 axis and a novel potential therapeutic target for the treatment of HBV-related HCC.

#### 2.1.5 LNC-DC

The key role of lnc-DC in regulating the differentiation, growth, and apoptosis of dendritic cells (DCs) has been investigated (Zhuang et al., 2018). Lnc-DC is a specific group leader ncRNA in DCs. The researchers isolated peripheral blood mononuclear cells for culture and induced DCs, which they then cocultured with HepG2.2.15 cells secreting HBV to detect lnc-DC changes. The expression of TLR9, p-STAT3 and SOCS3 were detected with qPCR and Western blotting. They analyzed cell proliferation using the 3-[4,5-dimethylthiazol-2-yl]-2,5 diphenyl tetrazolium bromide (MTT) assay and examined the cell cycle and apoptosis. lnc-DC knocked down pSTAT3, TLR9, and SOCS3 levels, demonstrating the involvement of TLR9/STAT3 signals. HBV DNA is regulated by lnc-DC and TLR9 signaling in DCs. This work elucidates the role of lnc-DC in the growth and immune response of DCs, potentially identify new mechanisms behind lnc-DC and immune responses in HBV infection.

#### 2.1.6 DLEU2

DLEU2 is a lncRNA expressed in the liver and upregulated in human HCC. As reported HBx binding to the promoter of DLEU2, which has enhanced the transcription of DLEU2, and induced the accumulation of DLEU2 in hepatocytes (Salerno et al., 2020). The level of HBV replication depends on the total number of cccDNA molecules and the amount of HBV RNA transcribed from each cccDNA. The cccDNA transcription is regulated by epigenetic modifications of cccDNA-bound histones by viruses, cellular proteins, and inflammatory cytokines. LncRNA reshapes chromatin by directing chromatin modification complexes toward their target loci. EZH2, a major cell H3K27 methyltransferase, catalyzes the addition of methyl to lysine 27 of histone H3 (H3K27me3). Nuclear DLEU2 binds directly to the histone methyltransferase enhancer (EZH2). EZH2 is the catalytic active subunit of the PRC2 complex. The co-recruitment of HBx and DLEU2 on cccDNA replaces EZH2 on viral chromatin and promotes viral transcription and replication. DLEU2 HBx binding to the target host promoter inhibits EZH2 and activates EZH2/PRC2 target genes in HBV-infected cells and HBV-related HCC.

#### 2.1.7 LncRNA H19

**Figure 2:**
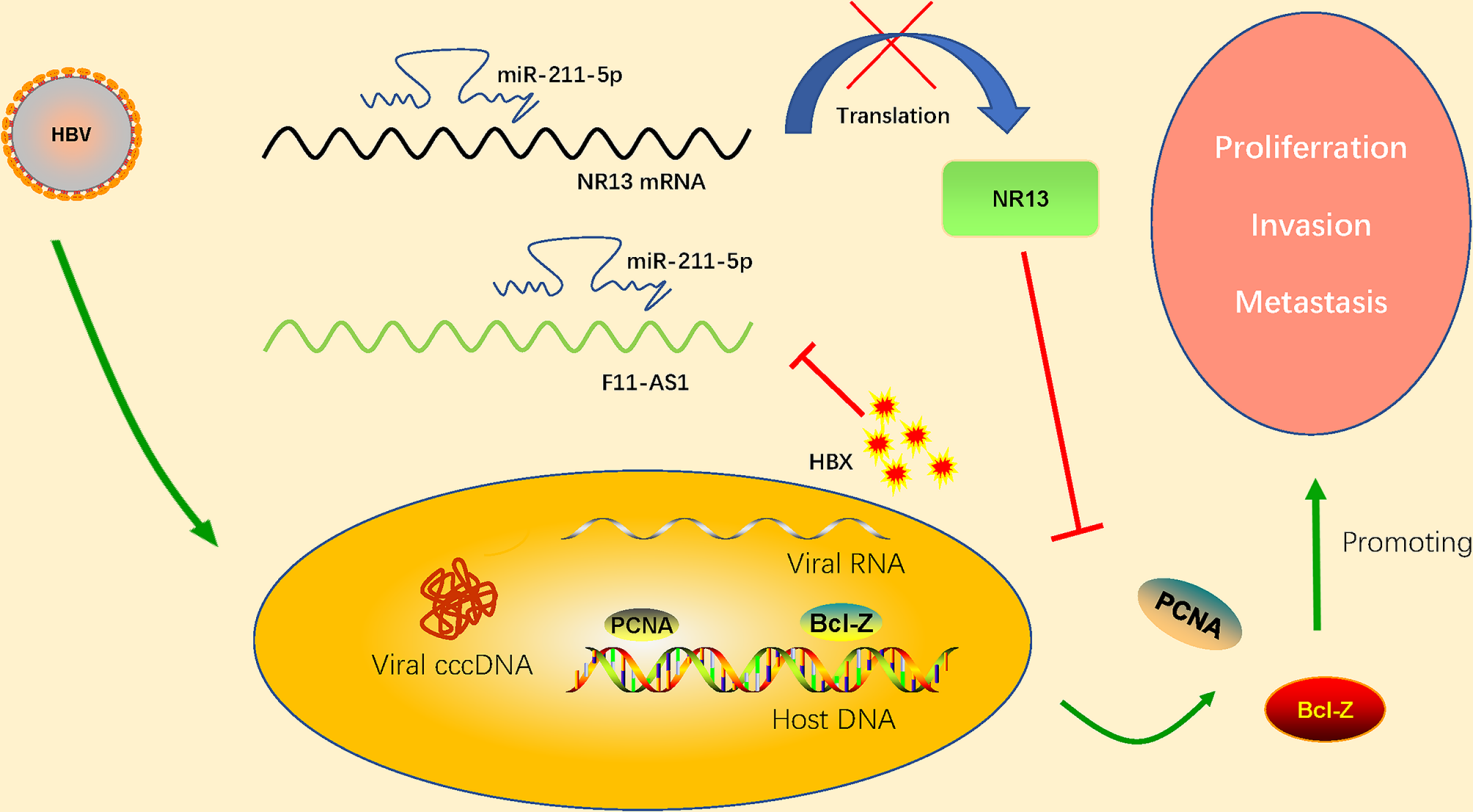
HBx overexpression upregulates expression of lncRNA H19 and miR-675, thus inhibiting expression of PPARα and further activating the Akt/mTOR signaling pathway. The H19/miR-675/PPARα/Akt/mTOR axis promotes HBx-induced hepatocyte injury, such as apoptosis, inflammation, oxidative stress, and energy metabolism remodeling.

LncRNA H19 belongs to a highly conserved cluster of imprinted genes and is expressed in many diseases characterized by inflammation and organ fibrosis. LncRNA H19 has been significantly upregulated in HBV-related HCC tissues (Li et al., 2019). Its upregulation is associated with the progression of HCC, and the expression of lncRNA H19 is correlated with tumor stage, distant metastasis and poor prognosis of HBV-related HCC. The results of Western blot showed that knockdown the lncRNA H19 could significantly decreased the expressions of series proteins, such as N-cadherin, Vimentin, β-catenin and MMP-9 which are crucial in EMT pathway. This result indicated that lncRNA H19 might regulate HBV-related HCC *via* EMT pathway. In addition, microRNA-22 has been found regulated by lncRNA H19 in HBV-related liver cancer. The abnormal expression of microRNA-22 is closely related to the proliferation, invasion, and metastasis of various malignancies. The study found that microRNA-22 expression is downregulated in HCC tissue and HCC cells. The role and underlying molecular mechanisms of the H19/miR-675 axis in HBx-induced hepatocyte injury *in vitro* has been identified (Liu et al., 2019). The data suggested that the H19/miR-675 axis may be an effective therapeutic target to protect the liver from damage caused by HBV infection. miR-675 direct targets the PPARα mRNA 3′-UTR. PPARα is closely related to the regulation of immune response, inflammatory response and energy metabolism. The H19/miR-675/PPARα axis regulates HBx-induced hepatocyte injury and energy metabolism remodeling, which is related to Akt/mTOR pathway. PPARα knockdown partially reversed the downregulation of H19 or miR-675 inhibition (Ge et al., 2019). These results indicate that the H19/miR-675/PPARα axis is associated with the regulation of Akt/mTOR signals in HBx-induced cell damage. The regulation mechanism of lncRNA H19 has shown in the **Figure 2**.

#### 2.1.8 Small Nucleoli RNA Host Gene 20 (SNHG20)

LncRNA SNHG20 acts as a tumor promoter in many cancers. LncRNA SNHG20 is upregulated in HBV-associated HCC cells (Tu et al., 2019). SNHG20 expression in hepatoma cells is positively correlated with HBx protein, and HBx-SNHG20 is involved in regulating the proliferation and apoptosis of hepatoma cells. In addition, SNHG20 interacts with PTEN to negatively regulate PTEN protein levels. A loss of PTEN leads to activation of the PI3K-Akt pathway or Jun-N kinase pathway, thus playing a key role in promoting carcinogenesis. PTEN protein is inhibited in HBV-positive HCC cells compared to HBV-negative ones; moreover, PTEN proteins promote HBx inhibition in positive HCC cells in a dose-dependent manner. In addition, HBx inhibits HCC apoptosis and promotes the proliferation of HCC cells by downregulating PTEN and reducing apoptosis in hepatoma cells. LncRNA SNHG20 is upregulated in the tumor tissue of HBV-positive HCC patients and HBV-positive cells, and HBx SNHG20 promotes the proliferation of HCC cells and reduces apoptosis in HCC cells by downregulating PTEN. This study demonstrates the interaction between SNHG20 and PTEN and the role of HBx/SNHG20/PTEN axic in HBV-related HCC. Potential targets are identified for the prevention and treatment HBV-related HCC.

#### 2.1.9 SAMD12-AS1

A latest study showed that upregulation of SAMD12-AS1 in HCC cells reduces p53 stability through the NPM1-HDM2-p53 axis, thus affecting cell proliferation and apoptosis (Liu et al., 2019). Functional analyses showed that SAMD12-AS1 promotes cell proliferation and inhibits apoptosis. Furthermore, it interacts with NPM1, which plays an important role in regulating rDNA transcription, and interacts with HDM2 to control the stability of p53. The interaction between SAMD12-AS1 and NPM1 reduces its association with E3 ligase HDM2, thus enhancing the interaction between HDM2 and p53 and promoting ubiquitin-mediated p53 degradation. As p53 is a tumor suppressor that is deregulated in different types of tumors, the negative correlation between SAMD12-AS1 and p53 stability suggests that SAMD12-AS1 may be a prognostic marker for HCC and other types of tumors. HBx-upregulated SAMD12-AS1 interacts with NPM1 and competes with NPM1 interactions with E3 ligases, leading to reduced p53 stability, which promotes cell proliferation and tumor growth.

Referring the studies listed above, lncRNAs regulating the proliferation of HCC cells in various of ways,which may concluding control the stability of p53 to make it more easy to be denatured and interact with HBx to downregulate the expression of PTEN, or co-recruitment both HBx and DLEU2 on the HBV cccDNA to replaces EZH2 on viral chromatin and promotes viral transcription and replication which may enhance the proliferation of HCC cells. In addition, HCC-related lncRNAs also affects the proliferation of HCC cells by regulating the expression of a sires of key genes such as TLR9, PTEN and p-STAT3 or other downstream genes in the pathway. Furthermore, lncRNAs are frequently dysregulated in HBV-related HCC and HBV-expressing/HBx-expressing cells, so it is reasonable that the co-action of HBx and LncRNAs plays a crucial factors in the regulation of the HCC cells proliferation and also has present a new prospective to find new regulation mechanisms. Relevant cases are scarce, and the specific regulatory mechanism remains to be studied.

### 2.2 LncRNAs Inhibit HCC Proliferation

#### 2.2.1 SEMA6A-AS1

SEMA6A-AS1 is a newly discovered antisense RNA of downregulated signaling protein 6 in L02/HBx cell lines. Yu et al. (2020) showed for the first time that a decrease in SEMA6A-AS1 expression is positively correlated with OS in HBV-related liver cancer. Decreased expression of SEMA6A-AS1 is associated with a poor prognosis in patients with HBV-related HCC. A number of antisense lncRNAs are often out of balance in liver cancer. By regulating its antisense protein-coding counterpart SEMA6A to promote liver cancer, SEMA6A-AS1 may be involved in the occurrence and development of HCC. SEMA6A is a transmembrane protein, originally described as a ligand, that mediates axonal orientation during the development of the central nervous system. As an inhibitor of tumor angiogenesis, SEMA6A regulates vascular development by regulating signal transduction of endothelial growth factor receptor (VEGFR)-2. In addition, SEMA6A expression is increased in renal cell lines, and recombinant soluble racemic cell domains inhibit basic fibroblast growth factor (bFGF/VEGF) and tumor cell-induced neovascularization. The SEMA6A-AS1 template sequence on human chromosome 5 consists of three exons of a total length of 967 nucleotides. SEMA6A-AS1 expression is decreased in HBV-related liver cancer. SEMA6A-AS1 may inhibit HBV-related liver cancer through RNA hybridization with SEMA6A mRNA and thus regulate SEMA6A expression.

#### 2.2.2 F11-AS1

**Figure 3:**
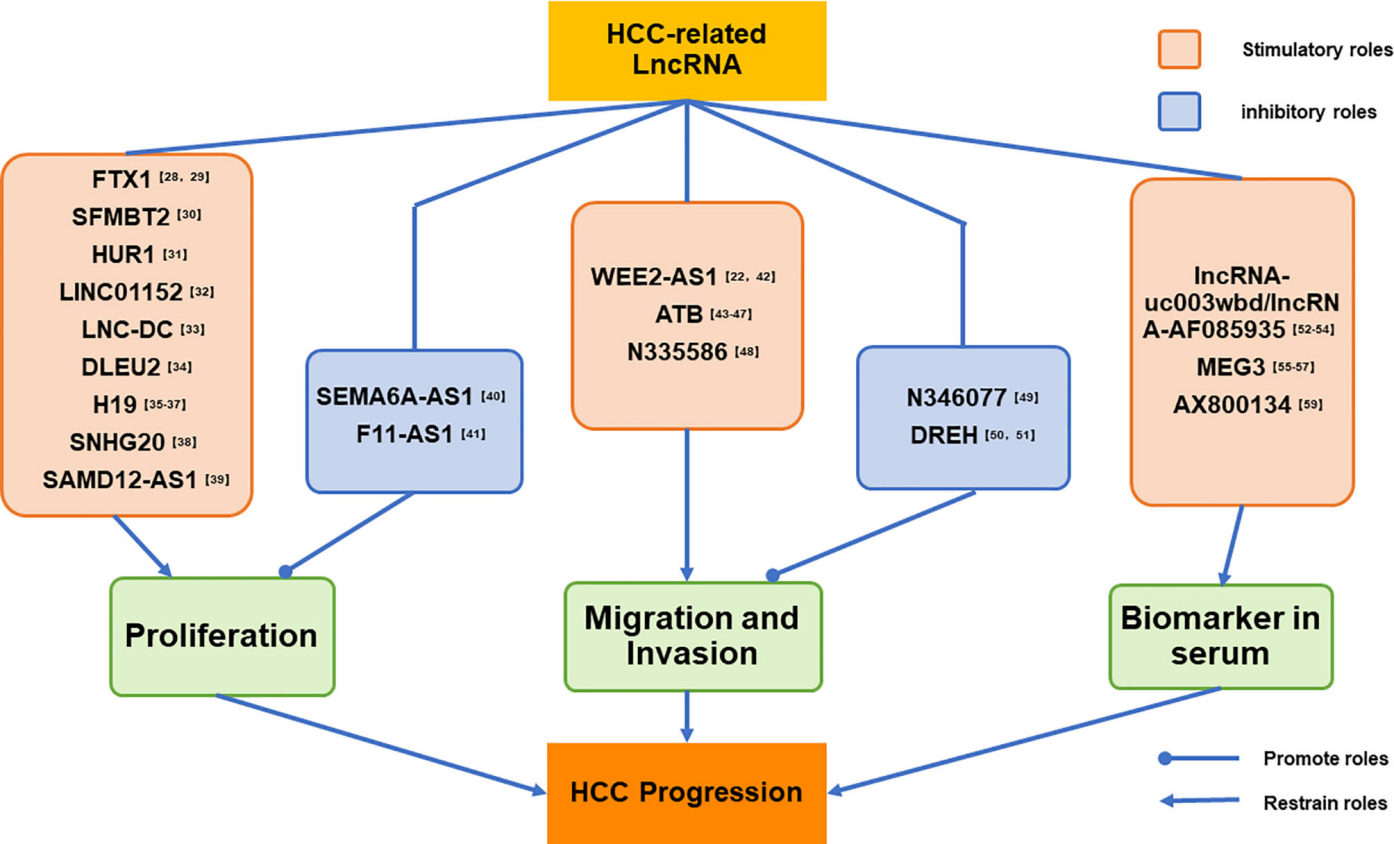
HBV-encoded HBx protein inhibits expression of lncRNA F11-AS1. lncRNA F11-AS1 upregulates NR1I3 by binding to miR-211-5p. The downregulation of lncRNA F11-AS1 caused by HBx protein weakens its ability to bind to miR-211-5p, thus reducing expression of NR1I3. As a result, the proliferation, migration, and invasion of HBV-related HCC cells are enhanced, but apoptosis is decreased. It is important to note that lncRNA F11-AS1 may enhance expression of NR1I3 by acting as the ceRNA of miR-211-5p, ultimately hindering the development of HBV(+) HCC.

The decreased expression of lncRNA F11-AS1 caused by HBx proteins is suspected to be associated with a poor prognosis in patients with HBV-associated liver cancer. Deng et al. (2020) confirmed that lncRNA F11-AS1 upregulates expression of NR1I3 by combining with miR-211-5p. microRNA-211 (miR-211) is closely related to the progression and development of human cancer. Deng et al. (2020) found that lncRNA F11-AS1 may regulate downstream gene expression by binding to miR-211-5p. In addition, they confirmed that nuclear receptor subfamily 1I group 3(NR1I3; also known as CAR) is the target gene of miR-211-5p. This study provides a complete set of regulatory pathways that lncRNA F11-AS1 interacts with miR-211-5p to upregulate NR1I3, thus hindering the development of HBV-related HCC. However, HBV-encoded HBx protein inhibits F11-AS1 expression and weakens its ability to bind to miR-211-5p, which may reduce NR1I3 expression. This results in the proliferation of HBV-related HCC cells and enhanced migration and invasion (apoptosis is reduced). LncRNA F11-AS1 overexpression may enhance NR1I3 expression by acting as a miR-211-5p’s ceRNA, eventually hindering the development of HBV(+)HCC. NR1I3 plays a fundamental role in regulating liver homeostasis and tumorigenesis, related to exogenous stress. It has also been considered as an important regulator of drug metabolism and cancer development. The important role of HBx has been proved that down regulate the expression of lncRNA F11-AS1 which cause a poor prognosis in HBV-related HCC patients. Although the lncRNA F11-AS1/miR-211-5p/NR1I3 axis participates in the progression of HBV-associated HCC by interfering with the cellular physiology of HCC. However, the clinical efficacy and potential application of the lncRNA F11-AS1/miR-211-5p/NR1I3 axis in the treatment of HBV-related liver cancer deserve further study to improve the overall prognosis of HCC patients. The regulation mechanism of lncRNA F11-AS1 in detail has shown in the **Figure 3**.

In the existing studies, there are not so much studies focusing on lncRNAs that inhibit the proliferation of HCC cells by regulating HCC-related pathways. But we can still realize that the way of lncRNAs regulating the target gene is acting as a ceRNA and bind to miRNAs, which can regulate pthe expression of downstream genes. The lncRNAs listed above mainly inhibit the proliferation of HCC cells by inhibiting the expression of key genes in HCC-related pathways such as NR1I3 and VEGF or BFGF. As there are still many HCC-related abnormal expression genes functions hasn’t been characterized, a large scale of relative researches need to launch to find more potential lncRNAs which take those genes as their own targets. Above all those factors, the role of lncRNA in more HCC related pathways needs further study.

## 3 LncRNAs Associated With HCC Migration and Invasion

The invasion and metastasis of liver cancer is an important factor affecting the therapeutic effect of liver cancer. Most of the lncRNAs are believed to regulate the migration, invasion and metastasis of HCC cells. For example, WEE2-AS1, ATB, N335586, N346077, DREH are related to the invasion and metastasis of HCC. WEE2-AS1, overexpressed in HBV-HCC, targets member 3 of the Fermitin family (FERMT3) to accelerate the proliferation, migration, invasion and cell cycle progression of HCC cells. ATB, activated by transforming growth factor-β(TGF-β), is closely related to the invasion and metastasis of hepatocellular carcinoma. N335586, increased in HBV-positive HCC tissues and cells, promoted the expression of its host gene CKMT1A through competitive binding of miR-924, thereby promoting the migration and invasion of HCC cells. N346077, one of the most down-regulated lncRNA in expression profile of HBV-HCC, inhibits invasion and migration of hepatocellular carcinoma cells. DREH can bind to intermediate silk protein vimentin and inhibit its expression, thus inhibiting tumor metastasis of HBV-HCC. Previous studies have shown that PI3K/Akt signaling pathway, JNK signaling pathway, TGF-β signaling pathway and NF-κB signaling pathway are the main pathways that affect the invasion and migration of HCC cells. LncRNAs can influence HCC metastasis by regulating the expression of certain epithelial mesenchymal transformation markers and blood vessel formation.

### 3.1 LncRNAs Promote HCC Migration and Invasion

#### 3.1.1 WEE2-AS1

HBx has been found to promote the progression of liver cancer by affecting lncRNA expression (Hu et al., 2019). LncRNA WEE2-AS1 is overexpressed in HBV-related HCC cells and tissue and is associated with a poor prognosis among HCC patients. Gain and loss of function experiments were performed to study the function of WEE2-AS1 in liver cancer. First, the researchers used 5′-RACE and 3′-RACE to obtain the full length of WEE2-AS1. Expression of lncRNA WEE2-AS1 in transfected hepatoma cell lines (HepG2, Huh7, SK-HEP-1, MHCC99H) was confirmed by immunofluorescence and real-time quantitative reverse transcription polymerase chain reaction (qRT-PCR). The results showed that ectopic expression of WEE2-AS1 promotes proliferation, colony formation, migration, invasion, and cell cycle progression in HCC cells but inhibits apoptosis.

Second, it was found that WEE2-AS1 regulates expression of Fermi family member 3 (FERMT3) in HCC and activates the PI3K/AKT/GSK3b signaling pathway. FERMT3 is the downstream target of WEE2-AS1. FERMT3, also known as Kinlin-3, is a gene closely related to tumorigenesis and development that regulates integrin activation and is deficient in many malignant tumors, such as chronic myelogenous leukemia, glioblastoma, and breast cancer (Lu et al., 2017). Further experiments showed that FERMT3 is upregulated by WEE2-AS1 and HBx and is positively correlated with HBV infection in liver cancer tissue. In addition, clinicopathological and survival analyses showed that overexpression of WEE2-AS1 is associated with hepatic vascular invasion, poor tumor differentiation, and poor prognosis. These results demonstrated that there is a preliminary HBx-WEE2-AS1- FERMT3 pathway which may serve as a therapeutic target for HBV-associated HCC.

#### 3.1.2 LncRNA-ATB

LncRNA-ATB, which is activated by transforming growth factor (TGF-β), is abnormally expressed in some tumors and is involved in tumor development (Tang et al., 2020). LncRNA-ATB is mainly distributed in the cytoplasm and the role of lncRNA-ATB in TGF-β signal transduction and HCC invasion and metastasis has been elucidated (Yuan et al., 2014). LncRNA-ATB, which is located on chromosome 14, plays an important role in regulating phenotypic transformation of human peritoneal mesothelial cells, HCC-related cirrhosis, and preeclampsia (Xiao et al., 2018). LncRNA-ATB upregulates ZEB1 and ZEB2 expression through competitive binding with the miR-200 family, thus inducing EMT and invasion. In addition, it induces IL-11 autocrine by binding to IL-11 mRNA and triggers STAT3 signaling, which promotes the histochemistry of metastatic tumor cells (Li and Kang, 2014). Overall, lncRNA-ATB promotes the invasion-transfer cascade reaction. These results indicate that lncRNA-ATB, a mediator of TGF-β signaling, may be a potential target for anti-HCC metastasis therapy. Zhang et al. (2020) investigated whether lncRNA-ATB is involved in HBx-mediated HCC. They detected expression of lncRNA-ATB in 26 HCC tissues and in a lentivirus-transfected HBx-HepG2 cell line. TGF-β activated expression of lncRNA-ATB, and expression of lncRNA-ATB and TGF-β increased and autophagy increased after HBx vector was transfected into HepG2 cells. Conversely, knocking out lncRNA-ATB or TGF-β inhibits this effect. Further regulatory mechanisms showed that lncRNA-ATB mainly performed its role by acting as a ceRNA to bind microRNAs like miR-200s. LncRNA-ATB could also regulate NF-κB, JAK/STAT3 and PI3K/AKT signaling pathways to perform its roles. In conclusion, HBx is closely related to carcinogenic lncRNA-ATB. HBx-induced autophagy upregulates expression of TGF-β and lncRNA-ATB. This may be a potential mechanism of HBV-induced HCC.

#### 3.1.3 N335586

N335586 was found to be one of the highest upregulated lncrnas in HBV-related liver cancers by deep sequencing (Fan et al., 2018). N335586 is differentially expressed in HBV-related HCC tissueand length of its transcript is about 300 nt. The overexpression vector pcDNA3/lncRNA n335586 (N335586) and the knockout plasmids pshR1-n335586 and pshR2-n335586 was constructed to explore the role of lncRNA n335586 in the malignant behavior of liver cancer cells. Expression of n335586 in HBV-positive liver cancer tissue and cells was significantly increased and was induced by HBV *in vitro*. Further mechanistic studies have shown that lncRNA n335586 promotes expression of its host gene CKMT1A by competitively binding with miR-924, thereby promoting the migration and invasion of liver cancer cells. In summary, the n335586/miR-924/CKMT1A axis is involved in the migration and invasion of liver cancer cells, which may help to explain the pathogenesis of HBV-related liver cancer.

Currently, most studies on lncRNA in the field of liver cancer tend to explore the influence of a single pathway on the growth, invasion and metastasis of liver cancer. However, few studies have found that a single lncRNA is involved in multiple pathways to co-regulate the progression, invasion and metastasis of liver cancer. As mentioned above, lncRNAs including WEE2-AS1, ATB, and n335586, were reported to promote HCC progression by promoting the invasion and metastasis of HCC cells. WEE2-AS1 accelerated the proliferation, migration, invasion and cell cycle progression of HCC cells by targeting Fermitin family member 3 (FERMT3) and then activating the PI3K/AKT/GSK3b signaling pathway. ATB, HBx−associated long non−coding RNA, activated by TGF−β promotes cell invasion and migration by inducing autophagy in primary liver cancer. n335586 promoted HCC cells migration and invasion through facilitating the expression of its host gene CKMT1A by competitively binding miR-924.The role of more lncRNAs in HCC related pathways needs further study.

### 3.2 LncRNAs Inhibit HCC Migration and Invasion

#### 3.2.1 N346077

The expression profiles of lncRNA in HBV-positive [HBV(+)HCC] and HBV-negative [HBV(–)HCC] liver cancer was studied (Fan et al., 2017). N346077 was found to be one of the most down-regulated lncRNA. High-throughput RNA sequencing technology (RNA-Seq), GO analysis, and KEGG pathway analysis were used to analyze cis- and trans-regulatory protein-coding genes to determine the lncRNAs involved in the progression of HBV-related HCC. N346077 encodes a 2609 bp transcript and it is located in the opposite strand of the mitochondrial ribosomal protein L23 (MRPL23) gene on chromosome 11. To investigate the role of this downregulated lncRNA in HCC cells, the researchers measured MTT, colony formation, cell migration, and invasiveness in HepG2 and QGY-7703 cells overexpressing n346077. The effectiveness of the n346077 overexpression plasmid was confirmed in the HepG2 and QGY-7703 cells. Transwell experiments showed that the migration and invasion ability of HepG2 and QGY-7703 cells was significantly inhibited after transfection with n346077, which indicates that n346077 inhibits the invasion and migration of liver cancer cells. However, the specific mechanism of n346077 inhibiting migration and invasion of HCC cells remains to be further studied.

#### 3.2.2 DREH

DREH is a highly conserved lncRNA on mammalian chromosome 17. It has two exons and does not contain polyadenylic acid. LncRNA DREH was identified that inhibits cell migration and plays a tumor-suppressing role in HBx-mediated liver cancer (Lv et al., 2017). They found that expression of DREH was significantly downregulated in HBV-related liver cancer tissue compared to adjacent liver tissue and was negatively correlated with the expression of HBx mRNA in HBV-related liver cancer tissue.

Huang et al. (2013) detected the expression profile of lncRNA in the liver of HBx transgenic mice and wild-type mice using lncRNA ChIP assay and qRT-PCR. In HBx transgenic mice, some lncRNA was dysregulated, and its expression was related to HBx. The frequent downregulation of DREH in the liver of HBx transgenic mice suggests that DREH may play a role in the development of HBV-related liver cancer. The effects of reduced DREH expression on cell proliferation, apoptosis, migration, and invasion were studied in BNL-CL2 and Hepa1-6 mouse hepatocyte cell lines. Expression of DREH was inhibited by RNA interference. Compared to the negative control, inhibition of DREH not only enhanced the cell proliferation effect but also promoted the migration and invasion activity of liver cancer cells. DREH can inhibit the growth and metastasis of liver cancer *in vivo* and *in vitro*, and play a cancer suppressor role in the development of HBV-HCC. DREH can bind to intermediate filament protein vimentin and inhibit its expression, thus changing the normal cytoskeleton structure and inhibiting tumor metastasis.

The specific mechanism by which lncRNAs inhibit the invasion and metastasis of liver cancer still needs to be further explored, and a large number of related biological functions and molecular mechanisms also need to be discovered. As described above, n346077, one of the most down-regulated long non-coding RNAs in transcriptome analysis of hepatitis B virus-associated hepatocellular carcinoma, has been proved to inhibit migration and invasion of hepatocellular carcinoma cells, but the specific mechanism needs to be further studied. DREH, which plays a key role in hepatocellular carcinoma, inhibits vimentin expression by acting as a tumor suppressor and inhibits HCC growth and metastasis *in vitro* and *in vivo*. The invasion and metastasis of liver cancer is a complex process with the interaction of multiple factors. To screen specific lncRNAs related to liver cancer metastasis, study its biological function and molecular mechanism, and further explore the relationship between lncRNAs involved in multiple signaling pathways, will make a great contribution to further revealing the molecular mechanism in the process of invasion and metastasis of liver cancer.

## 4 Biomarkers for HBV and HCC Screening

In oncology, biomarkers have diagnostic value, prognostic value and predictive value. Sensitive and specific biomarkers are particularly necessary in clinical precision medicine. Biomarkers can also serve as potential targets for drug design. Integrating biomarker data through bioinformatics can also expand our understanding of disease-related biological pathways and regulatory mechanisms. A variety of RNAs have been used as tumor biomarkers, of which lncRNA is the most widely studied. In recent years, many important functional RNAs that do not encode proteins have been discovered, some of which are also used as biomarkers, for example, the types of long non-coding RNAs we reviewed above are mainly concentrated at the tissue and cellular levels as biomarkers for HBV-HCC. Meanwhile, a large number of studies have shown that lncRNA plays an important role in gene expression regulation, cell growth, proliferation, apoptosis and cell communication. Current studies have also found that lncRNA expression is dysregulated to varying degrees in a variety of tumors, and is closely related to biological processes such as tumor genesis, development, invasion and migration. Studies have found that lncRNA, due to its abnormal expression in HCC and its specific molecular functions, is found to be a molecular marker for the diagnosis of HCC or a prognostic indicator. Some lncRNAs have been found to be dysregulated in liver cancer and have been shown to have clinical potential as diagnostic biomarkers and therapeutic targets. Serum lncRNAs can also be used as a new potential biomarker for the diagnosis of HBV-positive hepatocellular carcinoma, such as AX800134, MEG3, lncRNA-UC003WBD/lncRNA-AF085935. Abnormal expression of these lncRNAs is significantly associated with carcinogenesis, metastasis or prognosis. AX800134, as serum long non-coding RNA, can be used to diagnose HBV-positive HCC. Overexpression of AX800134 is a carcinogen in HCC, and its upregulation is associated with viral product HBx and chronic inflammation. LncRNA-MEG3 has been proved to be a serum marker for the diagnosis of chronic hepatitis B (CHB), improving the efficacy of early diagnosis and treatment. LncRNA UC003WBD and AF085935 can be used to distinguish liver cancer from hepatitis B and healthy people by analyzing their diagnostic value by analyzing lncRNA specifically expressed in blood. These results indicate that the above lncRNAs have great diagnostic value. The detection of serological tumor markers is of great significance for the early diagnosis and curative effect observation of hepatocellular carcinoma.

### 4.1 AX800134

AX800134 is a lncRNA with a length of 627 nucleotides. The serum long non-coding RNA AX800134, were used to diagnose HBV-positive HCC. Wang et al. (2015) used lncRNA chips to detect differential expression of lncRNA in HBV-positive HCC tissue and corresponding paracancerous tissues and found that lncRNA AX800134 was upregulated in HBV-infected HCC patients. The logistic regression model was constructed using training queues and verified by independent queues. The accuracy of diagnosis was evaluated by the area under the recipient’s operating characteristic curve (AUC). The expression of AX800134 can accurately diagnose HBV-positive HCC(AUC value of training cohort was 0.9494 and AUG value of validation cohort was 0.9491). In this lncRNA microarray study, AX800134 was identified as a new potential biomarker for the diagnosis of HCC.

In the study of lncRNA microarrays above, AX800134 was identified as a potential new biomarker for the diagnosis of HCC, especially in patients with AFP ≤ 400 ng/mL. Another study further validated lncRNA AX800134 expression in hepatitis B virus-associated hepatocellular carcinoma and the association between AX800134 upregulation and HBV-induced HCC and revealed its specific mechanism of action. Zuo et al. (2018) studied the effects of AX800134 on the growth and survival of liver cancer cells, analyzing the relationship between its upregulation and HBV infection. The results showed that HBV viral protein HBx directly induces expression of AX800134 in HepG2 cells, and proinflammatory cytokine TNFα enhances expression of AX800134; these effects were reversed by the ROS scavenger PDTC (Pan et al., 2016). These findings indicate that NF-κB signaling is related to the induction of AX800134 by HBx and TNFα. In addition, using siRNA to interfere with silencing AX800134 can significantly inhibit the growth and invasion of HBx-expressing HepG2 cells. These results show that high expression of AX800134 is a carcinogen for liver cancer. The clinical significance of this study is that it clarifies the development of new biomarkers and treatment strategies for diagnosing HBV-related HCC.

### 4.2 LncRNA-MEG3

LncRNA-MEG3 is a tumor suppressor gene mainly regulated by epigenetics. It is located on human chromosome 14q32.2 and is widely expressed in normal tissue (Song et al., 2019). LncRNA-MEG3 has multiple biological functions in different diseases. For example, it competes with the ceRNA of miR-181a to regulate cell proliferation, migration, and infiltration in gastric cancer (Peng et al., 2015). Liu et al. (2016) found that expression of lncRNA-MEG3 was significantly higher in tissue infected with gallbladder cancer than in adjacent normal tissue. Ectopic overexpression of MEG3 effectively inhibits the growth of gallbladder cancer cells.

Recent studies have shown that lncRNA-MEG3 can play a diagnostic role as a serum biomarker in patients with hepatitis B complicated with liver fibrosis. Chen et al. (2019) studied whether lncRNA-MEG3 can be used as a serum biomarker for the diagnosis of CHB. First, qRT-PCR was used to detect serum lncRNA-MEG3 levels in CHB patients and healthy controls. Subsequently, CHB patients were divided into HBeAg-positive and HBeAg-negative groups according to HBV infection, and the relationship between lncRNA-MEG3 level and HBV was explored. Correlations between serum levels of lncRNA-MEG3 and liver fibrosis were also analyzed. The serum lncRNA-MEG3 level of CHB patients was lower than that of the healthy control group, which was negatively correlated with the degree of liver fibrosis. Survival analyses showed that the serum lncRNA-MEG3 level has important value for diagnosing the degree of liver fibrosis in patients with CHB. In addition, expression of α-SMA and COL1A1 gradually increased in a time-dependent manner, whereas expression of lncRNA-MEG3 mRNA was downregulated. *In vitro* experiments further confirmed that expression of lncRNA-MEG3 is related to the degree of liver fibrosis in patients with CHB. Serum lncRNA-MEG3 expression was low in patients with chronic hepatitis B, and was negatively correlated with the degree of liver fibrosis. Serum lncRNA-MEG3 may serve as a diagnostic biomarker for CHB and the specific mechanism needs to be further studied.

### 4.3 lncRNA-uc003wbd/lncRNA-AF085935

In a study by Lu et al. (2015), the expression profiles of two types of lncRNA (lncRNA-uc003wbd and lncRNA-AF085935) in serum of hepatocellular carcinoma and HBV patients and their potential clinical value for distinguishing HBV patients from healthy specimens were discussed. Serum lncRNA-uc003wbd and lncRNA-AF085935 levels were up-regulated in HBV-positive HCC patients and controls. The serum lncRNA-uc003wbd and lncRNA-AF085935 levels of patients with AC/CC genotype and AG/GG genotype were lower than those of patients with AA genotype. LncRNA levels in serum samples from HBV patients, HCC patients and healthy controls were detected by real-time quantitative reverse transcription-polymerase chain reaction (qRT-PCR) and statistical analyses were performed with GraphPad. The three groups were distinguished according to their receiver operating characteristic (ROC) curves for each group. Levels of lncRNA-uc003wbd and lncRNA-AF085935 were significantly increased in the serum of HCC patients and HBV patients compared to the normal control group. These results show that both lncRNA-uc003wbd and lncRNA-AF085935 can be used as potential biomarkers for HCC and HBV screening. However, the specific mechanism of lncRNA-uc003wbd and lncRNA-AF085935 in hepatitis B virus-associated liver cancer has not been explored.

## 5 Conclusion and Prospects

In recent years, with the in-depth development of research, Evidences have show that lncRNAs can regulate gene expression at epigenetic, transcriptional and post-transcriptional levels, participate in a variety of important regulatory processes such as genomic imprinting, chromatin modification and transcriptional activation, and play a complex and precise regulatory role in development and gene expression. Studies have confirmed that lncRNAs are involved in tissue carcinogenesis, metastasis and cell proliferation, such as some lncRNAs are dysregulated in HBV-related HCC and HBV/HBx-expressing hepatocytes (Shi et al., 2016). These lncRNAs play a role in multiple tumor biological processes, such as regulating the proliferation, migration and invasion of liver cancer.We have summarized recent research on HBV-related lncRNAs and the role of HBV/HBx in regulating lncRNA expression and its mechanism of action as seen **Table 1**. For example, Some lncRNAs can promote the proliferation of liver cancer such as FTX, SFMBT2, HUR1, LINC01152, LNC-DC, DLEU2, H19, SNHG20, SAMD12-AS1. At the same time, some lncRNAs can inhibit the proliferation of liver cancer such as SEMA6A-AS1, F11-AS1. Some lncRNAs also play a role in the invasion and metastasis of HCC, promoting the invasion and metastasis of liver cancer such as WEE2-AS1, ATB, N335586 and inhibiting the invasion and metastasis of liver cancer such as N346077, DREH. AX800134, MEG3, lncRNA-uc003wbd and lncRNA-AF085935. The mechanisms by which these lncrnas function are also different. LncRNA can play a role as a ceRNA, and influence the proliferation and migration of liver cancer through competitive binding with miRNA. LncRNA FTX can mediate the transcriptional regulation of TIM-3 gene through negative regulation of microRNA-545 expression, and participates in the inflammatory response process of hepatitis B cirrhosis. LncRNA H19 can regulate microRNA-22 expression in HBV-associated liver cancer, which is closely related to tumor proliferation, invasion and metastasis. F11-AS1 enhances NR1I3 expression by competitively binding miR-211-5p, ultimately impeding the development of HBV-HCC. LncRNA ATB induces EMT and invasion by competitively binding with miR-200 family and up-regulating the expression of ZEB1 and ZEB2. LncRNA N335586 promotes the expression of its host gene CKMT1A through competitive binding with miR-924, thus promoting the migration and invasion of HCC cells.These findings may promote new methods of lncRNA-based cancer treatment, but more work needs to be done before such treatments can be used in clinical settings. In summary, HBV-related lncRNAs are of great significance for the study of physiopathological mechanisms of liver cancer. As research on the role of lncRNAs in liver diseases increases, HBV-related lncRNA will find a broader appeal in the diagnosis and treatment of liver cancer.

**Table 1:** lncRNAs linked to HBV related HCC.

LncRNA	Target	Functions	Pathway	Expression in HCC cells	Refs
FTX	Tim-3 mRNA 3′UTR	Promote the proliferation and metastasis of HCC cells	regulating tim-3 expression and affects the secretion of various inflammatory factors	promoting	(Zhao et al., 2014; Liu et al., 2018)
SFMBT2	LncRNA-Y5	Promote the proliferation of HCC cells	inhibiting the expression of LncRNA-Y5	promoting	(Yılmaz Susluer et al., 2018)
HUR1	P53	Promote the proliferation of HCC cells	Interacts with p53 to inhibit its transcriptional regulation of downstream genes	promoting	(Liu et al., 2018)
LINC01152	IL-23	Promote the proliferation of HCC cells	The hbX-UCA1/EZH2-P27KIp1 axis combined with the promoter of IL-23 to up-regulate IL-23	promoting	(Chen et al., 2019)
LNC-DC	STAT3	Promote the proliferation of HCC cells	signal TLR9/STAT3	promoting	(Zhuang et al., 2018)
DLEU2	PRC2	Promote HBV replication	DLEU2 binds directly to HBx and Zust homologue EZH2	promoting	(Salerno et al., 2020)
H19	microRNA-22	Promote the proliferation of HCC cells	The EMT pathway regulates the microrNa-22/H19/Mir-675/PPAR axis	promoting	(Li et al., 2019; Liu et al., 2019; Ge et al., 2019)
SNHG20	protein PTEN	Promote the proliferation of HCC cells and reduce the apoptosis of HCC cells	Activates the PI3K-Akt pathway or the Jun-N-terminal kinase pathway	promoting	(Tu et al., 2019)
SAMD12-AS1	NPM1、P53	Promote the proliferation of liver cancer cells and tumor growth	The stability of p53 was reduced by the NPM1-HDM2-p53 axis	promoting	(Liu et al., 2019)
SEMA6A-AS1	SEMA6AmRNA	Inhibit the emergence of HCC	With SEMA6AmRNA hybrid	inhibiting	(Yu et al., 2020)
F11-AS1	miR-211-5p	Inhibit the emergence of HCC	lncRNA F11-AS1/miR-211-5p/NR1I3 axis	inhibiting	(Deng et al., 2020)
AX8000134	Unknown	Enhance the growth and invasion	unclear	promoting	
WEE2-AS1	FERMT3	ccelerate the proliferation, migration, invasion and cell cycle progression of HCC cells.	unclear	promoting	(Hu et al., 2019)
ATB	MIR-200	LncRNA-miRNA/protein interaction,promote metastasis	unclear	promoting	(Yuan et al., 2014; Xiao et al., 2018; Tang et al., 2020)
n335586	MIR-924	promote HCC cells migration, invasion and EMT	unclear	promoting	(Fan et al., 2018)
n346077	MRPL23	suppress HCC cells invasion and migration	unclear	inhibiting	(Fan et al., 2017)
DREH	vimentin protein	inhibit HCC growth and metastasis	unclear	inhibiting	(Huang et al., 2013)

Although the strategy of diagnostic methods and treatment for hepatocellular carcinoma (HCC) is developing rapidly, such as new intervention chemotherapy, molecular targeted therapy and liver transplantation, but the liver cell cancer patients overall survival rates are still disappointing, urgent need to find new therapeutic targets and improved overall survival of HCC patients, hope for HCC patients. As lncRNAs are a new class of regulatory molecules, which regulate gene expression at the transcriptional, posttranscriptional or epigenetic levels, and affect the proliferation, apoptosis, invasion and metastasis of hepatocellular carcinoma cells, providing a new direction for the development and treatment of hepatocellular carcinoma. Although lncRNAs have attracted extensive attention as a research hotspot in recent years, many researchers have limited their exploration of the function of lncRNAs and their application in tumor diagnosis and treatment due to their biodegradability and instability of spatial structure. The role of lncRNA in HCC and the potential molecular mechanism still need to be further studied. Clarifying the relationship between lncRNA and the occurrence and development of HCC is particularly important for us to better understand the disease process and determine effective therapeutic targets and strategies.

## Author Contributions

Literature search, XW, MK, and CL. Figures, XW and TL. Study design, XH and XJ. Data collection, XW and MK. Writing, XW and MK. All authors contributed to the article and approved the submitted version.

## Funding

The authors declare that this study received funding from Scientific Research Foundation of Fuzhou University(GXRC-19025). The funder was not involved in the study design, collection, analysis, and interpretation of data, the writing of this article or the decision to submit it for publication.

## Conflict of Interest

Author XJ was employed by the company DAAN Gene.

The remaining authors declare that the research was conducted in the absence of any commercial or financial relationships that could be construed as a potential conflict of interest.

## Publisher’s Note

All claims expressed in this article are solely those of the authors and do not necessarily represent those of their affiliated organizations, or those of the publisher, the editors and the reviewers. Any product that may be evaluated in this article, or claim that may be made by its manufacturer, is not guaranteed or endorsed by the publisher.
